# Propranolol Relieves L-Dopa-Induced Dyskinesia in Parkinsonian Mice

**DOI:** 10.3390/brainsci10120903

**Published:** 2020-11-24

**Authors:** Ziqing Shi, Ian J. Bamford, Jonathan W. McKinley, Suma Priya Sudarsana Devi, Annie Vahedipour, Nigel S. Bamford

**Affiliations:** 1 Department of Pediatrics, Yale University, New Haven, CT 06510, USA; ziqing.shi.csu@foxmail.com (Z.S.); ianbamford@uchicago.edu (I.J.B.); jwmckinley@comcast.net (J.W.M.); sumapriya@gmail.com (S.P.S.D.); annie.vahedipour@yale.edu (A.V.); 2 Departments of Neurology and Cellular and Molecular Physiology, Yale University, New Haven, CT 06510, USA; 3 Department of Neurology, University of Washington, Seattle, WA 98105, USA

**Keywords:** Parkinson’s disease, striatum, acetylcholine, cholinergic, interneuron, β-adrenergic, receptor, levodopa, dopa-responsive, dystonia

## Abstract

Background: Parkinsonism is caused by dopamine (DA) insufficiency and results in a hypokinetic movement disorder. Treatment with L-Dopa can restore DA availability and improve motor function, but patients can develop L-Dopa-induced dyskinesia (LID), a secondary hyperkinetic movement disorder. The mechanism underlying LID remains unknown, and new treatments are needed. Experiments in mice have shown that DA deficiency promotes an imbalance between striatal acetylcholine (ACh) and DA that contributes to motor dysfunction. While treatment with L-Dopa improves DA availability, it promotes a paradoxical rise in striatal ACh and a further increase in the ACh to DA ratio may promote LID. Methods: We used conditional *Slc6a3^DTR/+^* mice to model progressive DA deficiency and the β-adrenergic receptor (β-AR) antagonist propranolol to limit the activity of striatal cholinergic interneurons (ChIs). DA-deficient mice were treated with L-Dopa and the dopa decarboxylase inhibitor benserazide. LID and motor performance were assessed by rotarod, balance beam, and open field testing. Electrophysiological experiments characterized the effects of β-AR ligands on striatal ChIs. Results: LID was observed in a subset of DA-deficient mice. Treatment with propranolol relieved LID and motor hyperactivity. Electrophysiological experiments showed that β-ARs can effectively modulate ChI firing. Conclusions: The work suggests that pharmacological modulation of ChIs by β-ARs might provide a therapeutic option for managing LID.

## 1. Introduction

Parkinsonism is caused by the lack of dopamine (DA) production or the death of DA-producing cells in the substantia nigra [1,2]. The reduction in DA availability to the motor striatum produces severe motor skill impairments, presenting as postural instability, rigidity, resting tremor, and bradykinesia. L-Dopa, the precursor of DA, increases DA availability to help restore motor function in patients with Parkinsonism. Despite its effectiveness, L-Dopa can produce a secondary hyperkinetic movement disorder called L-Dopa-induced dyskinesia (LID). LID occurs in 30% to 80% of patients with idiopathic Parkinson’s disease (PD) [3,4,5], as well as those with genetically-acquired Parkinsonism [6,7]. LID consists of choreic and dystonic movements that drastically reduce a patient’s quality of life [8]. The cause of LID is unknown but it is hypothesized to result from synaptic plasticity within the dorsal striatum [9], a brain structure that participates in motor learning and habit formation [10,11]. Recent evidence in mice indicates that LID may be caused by an imbalance between acetylcholine (ACh) and DA that is exacerbated by L-Dopa [12,13,14,15].

ACh plays a significant role in the pathogenesis of Parkinsonism. Before L-Dopa became available, anticholinergic drugs were sometimes used to treat PD. While clinically useful, their effect on the ACh system caused side effects because of their interaction with muscarinic ACh receptors [16]. Therefore, the ability to treat PD using either L-Dopa or anticholinergic drugs led to the hypothesis that a balance was needed between these two neurotransmitter systems [17,18] and suggested that an increase in ACh tone [19] or availability [20] accompanies DA deficiency. Striatal ACh is largely produced by tonically-active ACh-releasing interneurons (ChIs). ChIs receive excitatory input from the thalamus and are modulated by DA and ACh [21,22]. Although ChIs occupy just 2% of striatal neurons, their long and well-distributed axons can build tight connections with striatal spiny projection neurons that promote normal movements and establish motor learning [23,24].

Our recent work in mice has shown that under normal conditions, ACh and DA have a reciprocal relationship. As DA release decreases, ACh rises, and vice versa [13]. However, a progressive decline in striatal DA availability is accompanied by a smaller reduction in ACh. The reduction in striatal ACh is due to a decrease in production of hyperpolarization-activated cation (HCN) channel subunits that regulate the spontaneous firing rate of striatal ChIs as well as a reduction in choline acetyltransferase [13], the rate-limiting enzyme required for ACh production. While the release of both neuromodulators is reduced, ACh declines less than DA, so the ACh to DA ratio increases. When DA is released from residual axon boutons, ChI firing paradoxically increases, and the ACh to DA ratio increases further. Under these conditions, motor function becomes dependent on small changes in ACh, DA, and the ACh to DA ratio.

Since HCN channels, and thus the firing rate of ChIs, are regulated by cAMP, we hypothesized that LID might be relieved by using the non-selective β-adrenergic receptor (β-AR) antagonist 1-(Isopropylamino)-3-(1-naphthyloxy)-2-propanol ((−) propranolol). β1- and β2-ARs are distributed widely in the central nervous system, with a high concentration in the striatum [25]. β1-AR and β2-ARs are expressed on ChIs [25,26] where they enhance the production of cAMP [25]. Therefore, a β2-AR antagonist might stabilize ChI firing through a cAMP-dependent mechanism [27]. (-) propranolol has a 100× greater affinity for β-ARs than (+) propranolol [28], and it has been traditionally used to treat hypertension and cardiac disease, as well as neurological disorders including migraine, tremor, and tardive dyskinesia [29,30]. Clinical data has shown that propranolol may improve LID in patients with PD [31], but its use has gained little attention [4,32].

Here, we used L-Dopa-responsive, dopamine-deficient *Slc6a3^DTR/+^* mice as a model for LID to determine if β-ARs can modify ChI firing in the dorsolateral (DL) motor striatum and alleviate dyskinesia. *Slc6a3* encodes the DA transporter (DAT) that is expressed in most DA-producing cells. Expression of the diphtheria toxin (dT) receptor within the *Slc6a3* gene produces the *Slc6a3^DTR/+^* mutation and renders DA-producing cells highly susceptible to dT, which inhibits protein synthesis and leads to cell death [33]. Compared to primates or humans, wild-type (WT) mice lack dT-B receptors and so are 10^5^ times more resistant to dT [34]. We show that propranolol inhibits the spontaneous firing of ChIs in the DL stratum and reduces limb dyskinesia and hyperactivity in the subset of mice that develop LID.

## 2. Materials and Methods

### 2.1. Animals

All experimental procedures were approved by the Institutional Animal Care and Use Committee at Yale University (#2018-20055). Male and female mice (*n* = 73) were obtained from Jackson Labs (Bar Harbor, ME, USA) or bred in-house. Mice were housed together in a modified specific pathogen-free vivarium with a normal 12-hr light/dark cycle. Mice had access to food and water ad libitum, except during behavioral testing. For terminal procedures, mice were anesthetized using Beuthanasia (270 mg/kg i.p.) prior to sacrifice.

### 2.2. Experimental Design

We used *Slc6a3^DTR/+^* mice as a model of progressive DA deficiency [13]. Mice with the targeted expression of the human dT-receptor were generated by breeding WT C57Bl/6 mice with *Slc6a3^DTR/+^* mice, where one dT-receptor allele is expressed under the control of the *Slc6a3* gene that encodes the DAT [35]. 30-day-old *Slc6a3^+/+^* (*n* = 33) and *Slc6a3^DTR/+^* mice (*n* = 40) were treated with two doses of dT, 48 h apart. These mice were denoted WT(dT) and DAT(dT), respectively. For some studies, ChIs were fluorescently labeled by crossing *Slc6a3^+/+^* and *Slc6a3^DTR/+^* mice with ChAT-Cre^+/−^ (*B6.129S6-Chat*^tm2(cre)Lowl^*/J*; RRID:IMSR_JAX:006410) and tdTomato^+/−^ mice (*B6.Cg-Gt(ROSA)26Sor*^tm27.1(CAG-COP4*H134R/tdTomato)Hze^*/J*; RRID:IMSR_JAX:012567).

Prior investigations revealed that DAT(dT) mice develop Parkinsonism ~2 weeks following their first dT injection and have limited survival up to 30 days [13]. As such, mice slated for behavioral experiments were treated with L-Dopa and the dopa decarboxylase inhibitor benserazide on treatment week (TW) 1, 14 days following their first dose of dT. Thereafter, L-Dopa and benserazide were administered daily until the end of the experiment on TW 18. Low-dose propranolol was administered during TWs 10 to 12, and a higher concentration of propranolol was administered during TWs 13 to 15. A washout period without propranolol was provided on TWs 16 to 18.

Electrophysiology experiments in acute brain slices were performed to determine if progressive DA deficiency reduced the spontaneous firing of ChIs and modified responses to β-AR ligands in the absence of L-Dopa. Thirty-day-old *Slc6a3^+/+^* and *Slc6a3^DTR/+^* mice were treated with dT (as above) and were sacrificed 14 days after their first injection.

For all studies, the experimenters were blind to genotype. Sample-size estimates were determined by a power analysis based on prior work [23,36,37]. The studies included both male and female mice, but the number of mice prevented post hoc identification of outcome based on sex. During the electrophysiology experiments, “outliers” were defined as cells demonstrating an abrupt change in spontaneous activity or visual movement of the electrode across the surface of the cell. Outlier data were removed from all subsequent analyses. All electrophysiological recordings were replicated in four or more mice. The number of experimental repetitions is indicated in the results section.

### 2.3. Drug Administration

Diphtheria toxin (List Biological Laboratories, Campbell, CA, USA), with a half-life of ~18 h, was dissolved in 0.9% saline and administered intramuscularly [13]. L-Dopa (10 mg; Abcam #Ab142497), with an estimated half-life of 1 h, was dissolved in phosphate-buffered saline (PBS) (with 25 mg of ascorbic acid) for a concentration of 1 mg/mL [38]. The decarboxylase inhibitor benserazide (Abcam #Ab143181) was combined with the L-Dopa solution for a concentration of 0.48 mg/mL. Benserazide was used reduce some of L-Dopa’s untoward effects such as nausea. It may also prolong the half-life of L-Dopa. Propranolol HCl (Tocris # 0624), with a half-life of 3–6 h, was dissolved in 0.9% saline at concentrations of 0.4 mg/mL and 1.6 mg/mL. Propranolol and L-Dopa/benserazide were administered by separate i.p. injections. The vehicle consisted of an equal volume of 0.9% saline (0.01 mL/gm mouse). Unless specified in the text, chemicals and drugs were obtained from Sigma (St. Louis, MO, USA).

### 2.4. Behavior

Dyskinesia was scored by visual inspection each week and motor function was periodically assessed by rotarod, balance beam, and open field tests (Table 1 and Table 2).

#### 2.4.1. Dyskinesia Scoring

Mice were acclimated to the behavioral chamber for 90 min prior to treatment. Treatment was provided, and the animal was returned to the behavioral chamber for another 120 min. As the half-life of L-Dopa is ~1 h and that of propranolol is 3–6 h, the behavior of each subject was monitored by video recording for 1 min before treatment and then in 1 min intervals every 20 min for 100 min. Recordings were later reviewed and scored by a blinded experimenter. A modified dyskinesia scoring scale was developed, based on mice treated with 6-hydroxydopamine (6-OHDA) [39]. Two types of dyskinesia were identified and scored—limb dyskinesia was quick, repetitive, and uncontrolled limb movements; and general dyskinesia was shaking and involuntary movements of the arms. To separate the effects of novelty from the pharmacological effects of the drug, mice were acclimated to the entire procedure for 2 days prior to the weekly test.

#### 2.4.2. Motor Skill Learning

We used the rotarod test to detect alterations in basal ganglia-mediated motor coordination and learning [23]. We recorded the time that a mouse remained on a 10.5-cm rotating rod (Rotamex 4/8 system; Columbus Instruments, Columbus, OH, USA), accelerating from 5.4 rpm to 40 rpm over 4 min. The maximum time allowed for each trial was 240 s. A grip, where the mouse held on for one full rotation, was considered a fall. Mice were allowed three trials, with a 15-min inter-trial interval, and the results were averaged. To control for motor learning, the rotarod test was conducted before each new treatment phase and prior to their daily treatment. Mice were not exposed to the rotarod prior to the first test. The rotarod was disinfected with Clidox (VWR) following each trial, and no animals were tested during the inter-trial interval.

#### 2.4.3. Motor Coordination

Learning-independent motor coordination was measured using the balance beam [23]. Mice traversed a 60-cm-long, 15-mm-diameter cylindrical rod that was elevated 30 cm above a cushioned table. The mice were placed on one end of the beam and allowed to walk to the other side. Mice that fell were placed back on the beam at the position from which they had fallen and allowed to continue. The average velocity was recorded. The test was performed once a week and prior to their daily treatment. Mice were not exposed to the balance beam prior to the first test. The beam was disinfected with Clidox following each trial.

#### 2.4.4. Novelty Locomotion

We used animal activity monitor cages (San Diego Instruments, San Diego, CA, USA) to detect deficits in locomotion [23]. Four infrared beams separated by 8.8 cm that crossed the width of each chamber were connected to an International Business Machines computer, which recorded the number of times each beam was broken. Locomotor activity was measured by ambulations (two consecutive beam interruptions) summated over 5 min intervals. On each test day, animals were acclimated to individual activity chambers for 90 min. After the injection(s), mice were returned to their respective activity chambers. To avoid measuring an aversive response to handling and pain, and to capture the peak DA release [40], recordings were made for 80 min prior to treatment and for 60 min following the injection. Locomotor ambulations were measured three times each week, and results were averaged over the treatment interval. Following each experiment, locomotor chambers were cleaned with water, dried, and disinfected with Clidox.

### 2.5. Electrophysiology

Recordings of ChIs were obtained in a cell-attached configuration that allowed prolonged recordings of ChI firing [41]. Standard techniques were used to prepare 250-µm-thick slices for electrophysiology [42]. Brains were rapidly removed and mounted on a vibratome, submerged in ice-cold carbogenated (95% O_2_, 5% CO_2_) artificial cerebrospinal fluid solution (aCSF) containing (in mM): NaCl (124), KCl (5), NaHCO_3_ (26), NaH_2_PO_4_ (1.25), MgCl_2_ (2), CaCl_2_ (2), and glucose (10) at 35 °C (pH 7.2–7.4, 290–310 mOsm). Coronal sections containing the cortex and striatum were cut second to fourth frontal slice of the caudate-putamen (bregma, +1.54 to +0.62 mm) [43]. The brain slices were transferred to an incubating chamber with carbogenated N-methyl-d-glucamine (NMDG) -recovery solution, containing (in mM): NMDG (100), KCl (2.5), NaH_2_PO_4_ (1.2), NaHCO_3_ (30), 4-(2-hydroxyethyl)-1-piperazineethanesulfonic acid (HEPES) (20), MgSO_4_ (10), CaCl_2_ (0.5), and glucose (25) at 35 °C (pH 7.3–7.4, 300–310 mOsm). After 30 s, the slices were transferred to a holding chamber containing carbogenated aCSF at 35 °C. After 1 h, slices were placed on the stage of an upright Zeiss Axioskop FS microscope or an Olympus BX51WI microscope and bathed with continuously-flowing carbogenated aCSF (3 mL/min) at 35 °C.

ChIs were visualized in slices with the aid of fluorescent and infrared videomicroscopy coupled with differential interference contrast optics. ChIs were identified by tdTomato fluorescence and discriminated from striatal spiny projection neurons and GABAergic interneurons by their large soma (~25 µm), thick primary dendrites, autonomous firing in the absence of stimulation, high membrane capacitance, and stereotyped responses to current injection, as described [23,44]. At the end of each experiment, a whole-cell patch was obtained to confirm neuron type. Passive membrane properties were determined in voltage clamp mode by applying a depolarizing step voltage command (10 mV) and using a membrane test function integrated in the pClamp software. Electrodes were prepared from filamented borosilicate glass (King Precision Glass) on a P-97 electrode puller (Sutter Instruments, Novato, CA, USA). The electrodes (3–6 MΩ) were filled with an internal solution containing (in mM): KMeSO_4_ (119), MgCl_2_ (1), CaCl_2_ (0.1), HEPES (10), ethylene glycol-bis(β-aminoethyl ether)-N,N,N′,N′-tetraacetic acid (EGTA) (1), phosphocreatine (12), Na_2_ATP (2), and Na_2_GTP (0.7) (pH 7.2, 280–300 mOsm/L) [41,45]. Currents were Bessel filtered at 2 kHz and digitized at 50 µs using an IBM computer equipped with Digidata 1440 data acquisition and pClamp10.2 software (Molecular Devices, Silicon Valley, CA, USA).

### 2.6. Immunohistochemistry

Brains were incubated in 4% paraformaldehyde with 0.1 M phosphate-buffered saline (PBS; pH 7.4) for 24 h, 30% sucrose in PBS for 24 h, and then held in PBS (all at 4 °C). Next, 50 µm coronal sections were prepared with a vibratome and washed × 3 in PBS for 5 min. Sections were incubated in a blocking solution containing PBS with 2% bovine serum albumen and 0.2% Triton X-100 for 1 h before incubation with anti-choline acetyltransferase antibody (1:800 in blocking solution; AB144P, Millipore) overnight at 4 °C. Sections were washed × 4 in PBS for 5 min and incubated in Alexa Flour 488 antibody (1:200; A-11001, Invitrogen) for 2 h at room temperature. Following another round of washes in PBS (5 min × 4), sections were placed on a glass slide and cover-slipped with Fluoromount (Electron Microscopy Sciences) before imaging on a fluorescent microscope (Zeiss).

### 2.7. Statistical Analysis

Values given in the text and in the figures are mean ± standard error (SE). “*n*” represents the number of mice or cells indicated in the text. Differences in mean values were assessed by paired or un-paired 2-tailed *t*-tests. A Welch’s *t*-test was used for comparing unpaired data when there was a difference in variance between two population variances. The Holm-Sidak method with alpha = 0.05 was used for data requiring multiple comparisons. Repeated measures (rm) ANOVAs were used for data with multiple groups and a mixed-effects model was employed for data with missing values. Non-parametric data, as determined by an F-test, was compared using two-tailed Wilcoxon matched-pairs signed rank test (for paired groups) or the Kolmogorov–Smirnov (KS) test (for unpaired groups). ANOVA with Tukey’s multiple comparisons test was used to detect differences in groups due to treatment. Statistical analyses were performed with GraphPad Prism (v.8.3.1). Differences were considered significant if *p* < 0.05.

## 3. Results

### 3.1. L-Dopa Promotes Limb Dyskinesia in DAT(dT) Mice

**Figure 1:**
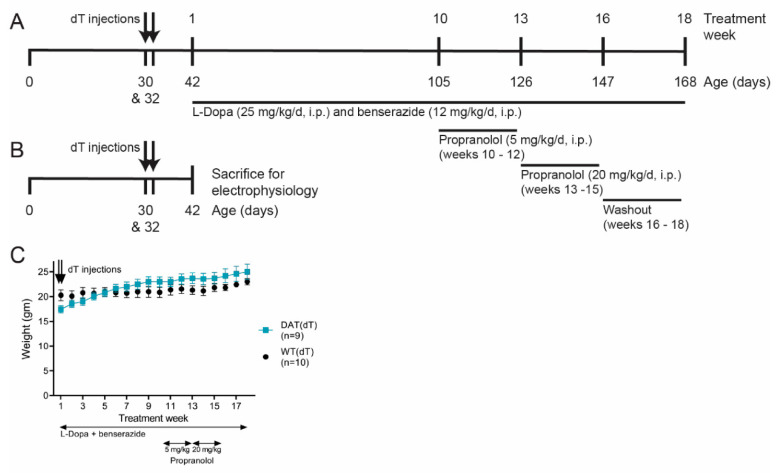
Dopamine transporter (DAT)(dT) mice as a model for DA deficiency. (**A**) Paradigm for testing the effects of propranolol on L-Dopa-induced dyskinesia (LID). (**B**) Paradigm for electrophysiology experiments. (**C**) DAT(dT) mice maintained weight over time.

We used L-Dopa-responsive, dopamine-deficient *Slc6a3^DTR/+^* mice to determine if propranolol, a β-AR antagonist, can modify LID, improve motor function, and stabilize ChI firing in the dorsolateral (DL) motor striatum. Male and female *Slc6a3^DTR/+^* received dT (50 μg/kg, i.m.; half-life ~18 h) at 30 and 32 days of life [13,33] and were termed DAT(dT) mice (Figure 1A). This treatment protocol produces a progressive reduction in striatal DA, ACh, and motor function [13]. Controls were similarly-treated *Slc6a3^+/+^* mice named WT(dT) mice. Under the treatment paradigm, dT had no effect on WT mice and genotype had no effect on motor performance [13]. As *Slc6a3^DTR/+^* transgenic mice became symptomatic ~2 weeks following their first injection of dT, mice received daily treatment with L-Dopa (25 mg/kg/day, i.p.) [38] and the peripherally-acting dopa decarboxylase inhibitor benserazide (12 mg/kg/day, i.p.) [46] beginning on day of life 42 (TW 1) and terminating on day 168 (TW 18). Mice used for electrophysiological experiments received dT at 30 and 32 days of life and were sacrificed 2 weeks later (Figure 1B). Treatment had no effect on the animal’s ability to feed, as DAT(dT) and WT(dT) mice demonstrated similar weight over the course of treatment (Figure 1C).

Dyskinesia were assessed weekly. We adapted and modified a dyskinesia scoring system used previously [5,39]—limb dyskinesia was repetitive leg movements whose severity depended on the frequency and number of limbs involved, while general dyskinesia included twitching and shaking of the trunk and the arms (Table 1). Motor function was periodically assessed by the latency to fall from an accelerating rotarod, the time required to traverse a balance beam, and ambulations in the open field (Table 2). Dyskinesia and behavioral measures were averaged over each treatment interval.

**Figure 2:**
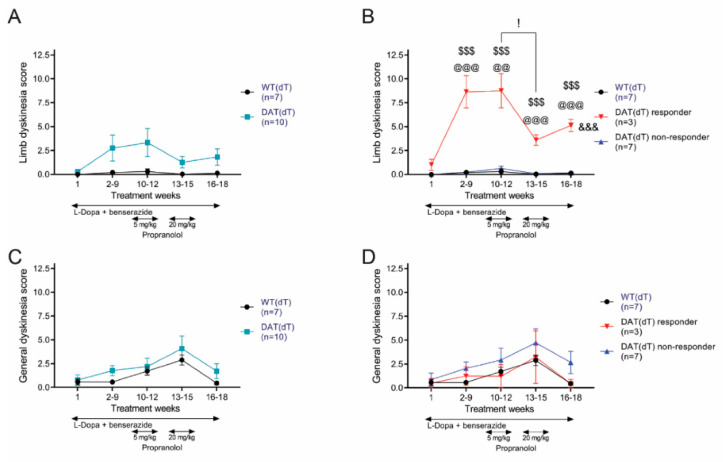
L-Dopa produced dyskinesia in DAT(dT) mice. (**A**) Limb dyskinesia was statistically similar in DAT(dT) and WT(dT) mice. (**B**) DAT(dT) responders manifested limb dyskinesia that was reduced by the higher dose of propranolol. (**C**) General dyskinesia was similar in DAT(dT) and WT(dT) mice. (**D**) DAT(dT) responders did not manifest enhanced general dyskinesia. For all figures, data are represented by mean ± SE. ^@@^ *p* < 0.01, ^@@@^ *p* < 0.001, *t*-test or KS test, DAT(dT) responder or DAT(dT) non-responder vs. WT(dT); ^$$$^ *p* < 0.001, *t*-test; DAT(dT)responder vs. non-responder mice; ^&&&^ *p* < 0.001, two-way rm-ANOVA, for interaction between groups and treatment.

Prior to receiving their first dose of L-Dopa and benserazide on TW 1, WT(dT) and DAT(dT) mice had similar limb dyskinesia scores (0.3 ± 0.2 for DAT(dT) (*n* = 10) vs. 0 ± 0 for WT(dT) (*n* = 7); *p* = 0.3, *t*-test; Figure 2A). Treatment with L-Dopa and benserazide produced a significant limb dyskinesia in a subset (30%) of DAT(dT) mice (*n* = 3) that were labelled as DAT(dT) responders, with behavior characterized by repeated jumping and rotating (8.6 ± 1.7 for DAT(dT) responders vs. 0.2 ± 0.1 for WT(dT); *p* < 0.001, *t*-test; Figure 2B; Supplementary Materials videos 1 and 2). Limb dyskinesia in DAT(dT) responder mice was much higher than other similarly-treated DAT(dT) mice, labelled DAT(dT) non-responders (0.2 ± 0.1; *n* = 7; *p* < 0.001, *t*-test, compared with DAT(dT) responders). Limb dyskinesia in DAT(dT) non-responders was similar to those in WT(dT) mice (*p* = 0.9, *t*-test). Like those with PD [3,4,5], only a subgroup of subjects developed LID.

### 3.2. Propranolol Reduces Limb Dyskinesia in DAT(dT) Mice

On TW 10, WT(dT) and DAT(dT) mice began receiving additional daily treatments of low-dose propranolol (5 mg/kg/d; i.p.), extending for 3 weeks (TWs 10 to 12). On TW 13, the daily dose of propranolol was increased to the target dose of 20 mg/kg/d; i.p. [47]. After three additional weeks of treatment, propranolol was stopped, providing a 3-week wash out (TWs 16 to 18). This treatment paradigm was used to establish dose-dependency on LID and motor activity. Low-dose propranolol had no effect, but the larger dose led to a sharp reduction of limb dyskinesia in the DAT(dT) responders (*p* = 0.04, ANOVA with Tukey’s multiple comparisons test; Supplementary Materials Videos 2 and 3). After propranolol treatment was discontinued, we observed no significant changes in dyskinesia scores across the treatment groups. Overall, treatment had a significant impact on limb dyskinesia in DAT(dT) responder mice, compared to WT(dT) mice (F_(4,32)_ = 22.96; *p* < 0.001, rm-ANOVA) and DAT(dT) non-responders (F_(4,32)_ = 24.41; *p* < 0.001, two-way rm-ANOVA, interaction between groups and treatment).

Measures of general dyskinesia were similar in DAT(dT) (0.8 ± 0.5) and WT(dT) mice (0.6 ± 0.3; *p* = 0.8, *t*-test) on TW 1 (Figure 2C). Treatment with L-Dopa and benserazide with or without propranolol had no effect on general dyskinesia scores from DAT(dT) mice (F_(4,60)_ = 0.5; *p* = 0.8, rm-ANOVA, interaction between groups and treatment). Similarly, general dyskinesia scores in DAT(dT) responder mice were similar to WT(dT) mice (F_(4,32)_ = 0.4; *p* = 0.8, rm-ANOVA) and to DAT(dT) non-responder mice (F_(4,36)_ = 0.14; *p* = 0.9, rm-ANOVA, interaction between groups and treatment; Figure 2D). 

### 3.3. Propranolol Had No Effect on Rotarod Performance

**Figure 3:**
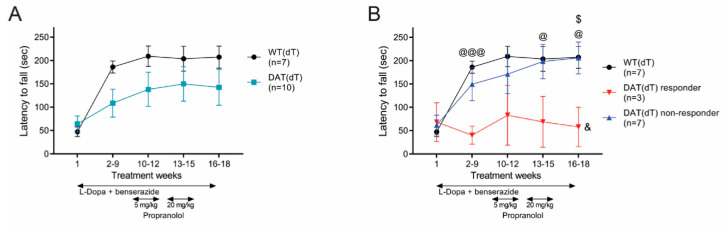
Rotarod performance in DAT(dT) mice. (**A**) The latency to fall from an accelerating rotarod was similar in DAT(dT) and WT(dT) mice. (**B**) DAT(dT) responders demonstrated a low latency to fall from the rotarod. ^@^ *p* < 0.05, ^@@@^ *p* < 0.001, *t*-test or KS test, DAT(dT) responder or DAT(dT) non-responder vs. WT(dT); ^$^ *p* < 0.05, *t*-test; DAT(dT) responder vs. non-responder mice; ^&^ *p* < 0.05, two-way rm-ANOVA, for interaction between groups and treatment.

We performed rotarod, balance beam, and locomotor tests on DAT(dT) and WT(dT) mice at the beginning and at the end of each TW to determine if propranolol might alter motor function. Rotarod performance of both groups increased over time, consistent with motor learning [23] (Figure 3A). On TW 1, the latency to fall from the rotarod of DAT(dT) mice (64 ± 17.1 s; *n* = 10) was similar to WT(dT) mice (47.5 ± 10.1 s; *n* = 7). Thereafter, there was a shorter, but non-significant latency to fall in DAT(dT) mice compared to WT(dT) mice (F_(1,18)_ = 3.46; *p* = 0.07, two-way rm-ANOVA; Figure 3B). On TW 1, the latency to fall from the rotarod of DAT(dT) responders (68 ± 41 s; *n* = 3), DAT(dT) non-responders (62 ± 21 s; *n* = 7), and WT(dT) mice (47 ± 10 s) was similar. During TWs 2 to 9, DAT(dT) responders had a shorter latency to fall than WT(dT) mice (40 ± 19 s vs. 186 ± 13 s for WT(dT); *p* < 0.001, *t*-test). Propranolol had no effect on rotarod performance in DAT(dT) responder or non-responder mice (*p* > 0.4, ANOVA with Tukey’s multiple comparisons test). Overall, DAT(dT) responders showed little motor learning and had a shorter latency to fall than either WT(dT) mice (F_(4,32)_ = 3.8; *p* = 0.02, two-way rm-ANOVA) or DAT(dT) non-responders (F_(2,36)_ = 3.6; *p* = 0.02, two-way rm-ANOVA, interaction between groups and treatment). Therefore, while DA replenishment rescued DAT(dT) mice, those subjects with LID demonstrated lower rotarod performance with and without propranolol.

### 3.4. High-Dose Propranolol Improves Balance-Beam Performance

**Figure 4:**
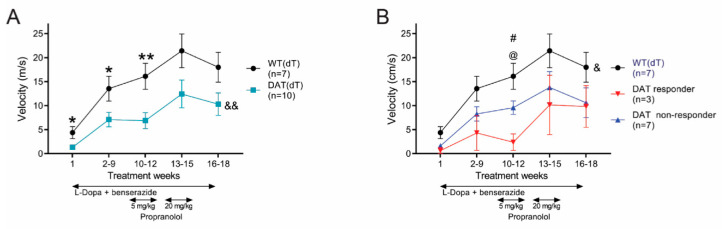
Balance beam performance in DAT(dT) mice. (**A**) DAT(dT) mice were slower on the balance beam. (**B**) DAT(dT) responders were slower on the balance beam. * *p* < 0.05, ** *p* < 0.01, *t*-test or KS test, DAT(dT) vs. WT(dT); ^@^ *p* < 0.05, *t*-test or KS test, DAT(dT) responder or DAT(dT) non-responder vs. WT(dT); ^#^ *p* < 0.05, *t*-test, DAT(dT) non-responder vs. WT(dt) mice; ^&^ *p* < 0.05, ^&&^ *p* < 0.01, two-way rm-ANOVA, for interaction between groups and treatment.

Next, we determined if propranolol could modify balance-beam performance. During TW 1, the velocity of DAT(dT) mice (1.3 ± 0.3 cm/s) was slower than WT(dT) mice (4.4 ± 1.3 cm/s; *p* = 0.01, *t*-test; Figure 4A). Over time, both groups showed improvement in performance, with DAT(dT) lagging WT(dT) mice (F_(1,15)_ = 9.1; *p* = 0.009, two-way rm-ANOVA). During TWs 2 to 9, the average velocity of DAT(dT) mice (7.1 ± 1.5 cm/s) was lower than WT mice (13.6 ± 2.6 cm/s; *p* = 0.03, *t*-test). Low-dose propranolol had no effect on DAT(dT) mice (6.9 ± 1.6 cm/s; *p* = 0.9, ANOVA with Tukey’s multiple comparisons test), which remained slower than WT(dT) mice (16.1 ± 2.7 cm/s; *p* = 0.01, *t*-test). High-dose propranolol increased the average velocity of DAT(dT) mice (12.4 ± 2.9 cm/s) and reduced the difference between DAT(dT) and WT(dT) mice (21.4 ± 3.5 cm/s; *p* = 0.08, *t*-test). The velocity of both DAT(dT) mice (10.3 ± 2.3 cm/s) and WT(dT) mice (18 ± 3.1 cm/s) was similar during the washout (*p* = 0.07, *t*-test). 

On TW 1, the balance beam velocity of DAT(dT) responders (0.6 ± 0.6 s; *n* = 3), DAT(dT) non-responders (1.6 ± 0.3 s; *n* = 7), and WT(dT) mice (4.4 ± 1.3 s; *n* = 7) were similar. However, over time the velocity of DAT(dT) responders (F_(1,8)_ = 5.41; *p* = 0.04, two-way rm-ANOVA) and non-responders (F_(1,13)_ = 6.9; *p* = 0.02, two-way rm-ANOVA) were both slower than WT(dT) mice (Figure 4B). The velocity of DAT(dT) responders was similar to DAT(dT) non-responders (F_(1,9)_ = 1.8; *p* = 0.2, two-way rm-ANOVA). With low-dose propranolol, DAT(dT) responders (2.4 ± 1.7 s) and non-responders (9.6 ± 1.4 s) were slower than WT(dT) mice (16.1 ± 2.7 s). Compared to low-dose propranolol, high-dose propranolol increased the velocity of both DAT(dT) responders (10.2 ± 6.2 cm/s) and non-responders (13.8 ± 3.3 cm/s) to reduce their differences with WT(dT) mice (21.4 ± 3.51 cm/s; *p* > 0.1, *t*-test). Therefore, L-Dopa-treated DA-deficient mice demonstrated preserved motor learning but manifested a lower balance beam performance and a modest improvement with high-dose propranolol.

### 3.5. Propranolol Improves Locomotor Hyperactivity

**Figure 5:**
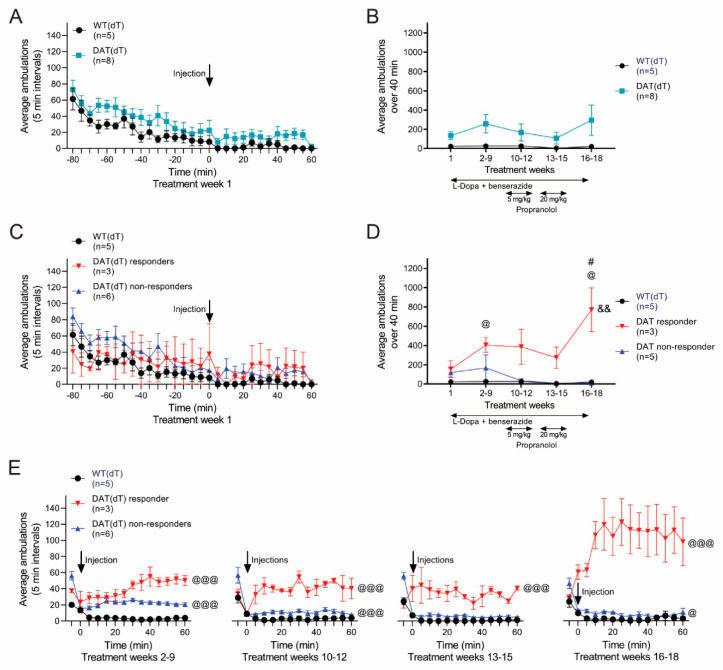
Locomotor ambulations are reduced in DAT(dT) responders. (**A**) Ambulations of DAT(dT) and WT(dT) mice were similar when measured on TW 1, before and following treatment (arrow) with L-Dopa and benserazide, (**B**) Ambulations in DAT(dT) and WT(dT) mice were similar across treatment weeks. (**C**) Ambulations in WT(dT) mice were similar to DAT(dT) responders and non-responders when measured on TW 1, before and after treatment. (**D**) DAT(dT) responder mice demonstrated a higher number of ambulations following treatment. Ambulations in WT(dT) and DAT(dT) non-responders were similar. (**E**) Average ambulations over time following treatment. ^@^ *p* < 0.05, ^@@@^ *p* < 0.001, *t*-test or KS test, DAT(dT) responder or DAT(dT) non-responder vs. WT(dT); ^#^ *p* < 0.05, *t*-test, DAT(dT) non-responder vs. WT(dt) mice; ^&&^ *p* < 0.01, two-way rm-ANOVA, for interaction between group(s) and treatment.

Next, we monitored locomotor activity in DAT(dT) and WT(dT) mice. Ambulations were counted for 80 min prior to treatment and for 60 min following. On TW 1, pre-treatment recordings revealed similar average ambulations in DAT(dT) (29 ± 3; *n* = 8) and WT(dT) mice (13 ± 3.5; *n* = 5; F_(1,11)_ = 1.78; *p* = 0.2, two-way rm-ANOVA), where habituation to the cage was manifest by a progressive reduction in ambulations over time (Figure 5A). Following the injection of L-Dopa and benserazide, the average number of ambulations in DAT(dT) mice (134 ± 41) was higher than those of WT(dT) mice (23 ± 15), but the results were not significant (*p* = 0.1, KS test; Figure 5B). Similarly, ambulations of DAT(dT) mice across the treatment groups were higher than WT(dT), but there was no significant difference (F_(1,11)_ = 3.65; *p* = 0.08, two-way rm-ANOVA).

Pre-treatment ambulations on TW 1 were similar in DAT(dT) responder mice (*n* = 3), DAT(dT) non-responder mice (*n* = 5), and WT(dT) mice (*n* = 5; Figure 5C). Treatment of DAT(dT) non-responder mice with L-Dopa and benserazide, with or without propranolol, had no effect on their locomotor ambulations, and their ambulations remained similar to WT(dT) mice (*p* > 0.1, KS test). However, with L-Dopa and benserazide DAT(dT) responder mice manifested a significant increase in their ambulations during TWs 2 to 9 (406 ± 76 for DAT(dT) responders vs. 27 ± 8 for WT(dT); *p* = 0.03, KS test) that reduced following high-dose propranolol (274 ± 111 for DAT(dT) responders vs. 5 ± 2 for WT(dT); *p* = 0.12, KS test; Figure 5D,E). There was a considerable increase in locomotor activity in DAT(dT) responders during the wash-out, rising to 771 ± 227 ambulations when receiving L-Dopa and benserazide alone (*p* = 0.03, KS test; compared with 21 ± 5 ambulations in WT(dT) mice). Treatment had a significant effect on the locomotor performance of DAT(dT) responders compared to either WT(dT) mice (F_(4,24)_ = 5.64; *p* = 0.002, two-way rm-ANOVA) or DAT(dT) non-responders (F_(4,24)_ = 4.37; *p* = 0.009, two-way rm-ANOVA, for interaction between groups and treatment). Together, the data suggest that the mice which develop limb dyskinesia manifest higher locomotor ambulations during treatment with L-Dopa and benserazide but have lower motor coordination and skill learning. Propranolol may improve limb dyskinesia and hyperactivity without adversely affecting motor performance.

### 3.6. β-Adrenergic Receptors Modulate ChI Firing

**Figure 6:**
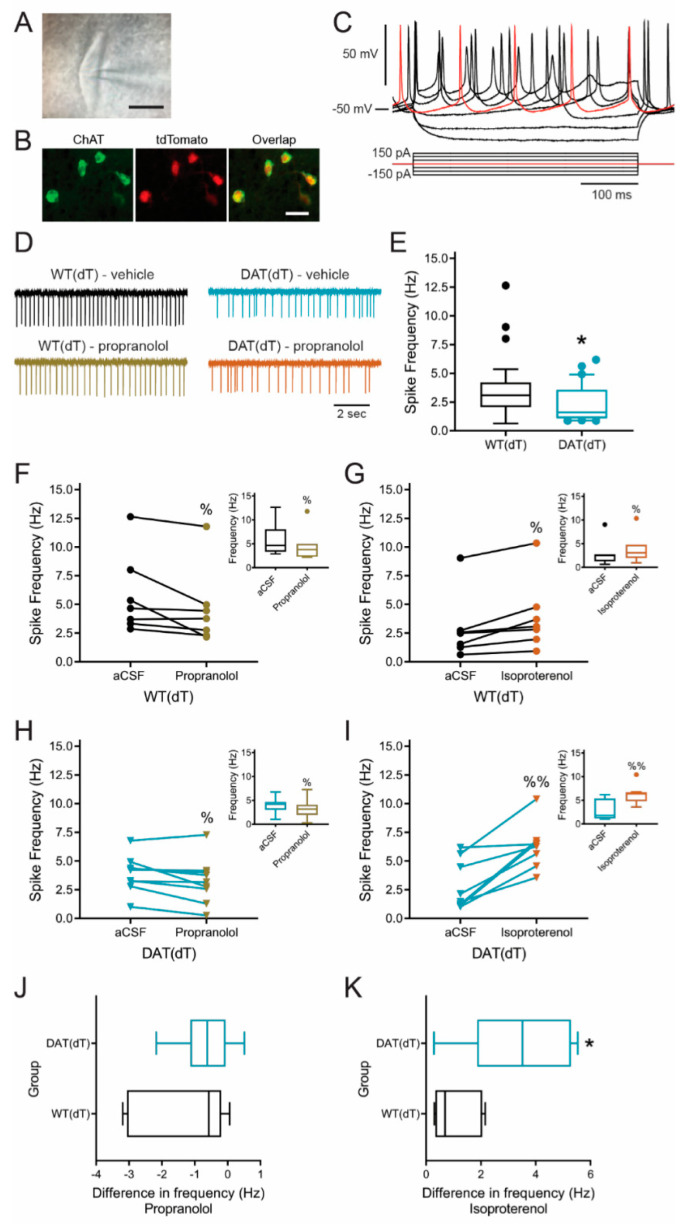
β1-ARs modulate ChIs in the DL striatum. (**A**) Cell-attached recording of a ChI. Scale bar, 10 µm. (**B**) Antibody-labeled ChIs in the striatum of ChAT-Cre^+/−^ x tdTomato^+/−^ mice. Scale bar, 20 µm. (**C**) Whole-cell recording in current clamp mode shows the typical ChI response (above) to applied current (below). (**D**) Representative traces of cell-attached recordings show that the firing rate was reduced in ChIs from DAT(dT) mice, compared to WT(dT) mice. Propranolol reduced firing in ChIs from both DAT(dT) and WT(dT) mice. (**E**) The firing frequency of ChIs in the DL striatum was lower in DAT(dT) mice than WT(dT) mice. Tukey box and whisker plot: The box extends from the 25th to 75th percentiles. The line in the middle of the box is plotted at the median. Whiskers are plotted at the 25th to 75th percentiles plus or minus 1.5 times the inter-quartile distance (the difference between the 25th and 75th percentiles). Circles are the calculated outliers. (**F**) Individual and cumulative (inset) plots show that propranolol reduced spontaneous firing in ChIs. The graph shows the response of individual cells while the box-plot inset demonstrates the median and centiles. (**G**) Individual and cumulative (inset) plots show that isoproterenol increased firing in ChIs from WT(dT) mice. (**H**) Individual and cumulative (inset) plots demonstrate that propranolol reduced firing in ChIs from DAT(dT) mice. (**I**) Individual and cumulative (inset) plots show that the firing rate of ChIs in DAT(dT) mice was enhanced by isoproterenol. (**J**) Graph compares the reduction in the ChI firing rate by propranolol in DAT(dT) and WT(dT) mice. (**K**) Graph compares the enhanced spontaneous firing in ChIs from DAT(dT) and WT(dT) mice during isoproterenol. * *p* < 0.05, *t*-test or KS test, DAT(dT) vs. WT(dT); ^%^ *p* < 0.05, ^%%^ *p* < 0.01, Wilcoxon signed rank test.

The mechanism underlying LID is unknown, but animal studies suggest that the disorder develops through an L-Dopa-dependent increase in ACh production that heightens an imbalance between ACh and DA [12,13]. Striatal ACh is predominantly produced by ChIs that are putatively regulated by β-ARs [25,26]. To examine the effect of β-AR on the spontaneous firing rate of ChIs in the DL striatum, we treated 30-day-old *Slc6a3^DTR/+^* (*n* = 30) and *Slc6a3^+/+^* (*n* = 26) mice with dT and obtained cell-attached recordings of ChIs 2 weeks following treatment (Figure 6A–C) [13]. Experiments in 6-week-old C57B/6 mice allowed comparisons with our prior work in dopamine-deficient mice [13,38].

The baseline firing rate of ChIs in slices from DAT(dT) mice (2.4 ± 0.3 Hz; range, 0.9 to 6.2 Hz; *n* = 30 cells) was lower than WT(dT) mice (3.7 ± 0.5 Hz; range, 0.6 to 12.6 Hz; *n* = 26; *p* = 0.03, Welch’s *t*-test; Figure 6D,E). ChI firing in WT(dT) mice decreased in response to bath application of the β-AR antagonist propranolol (1 µM [48]; 4.6 ± 1.2 Hz vs. 5.8 ± 1.3 Hz in vehicle; *n* = 7; *p* = 0.03, Wilcoxon signed rank test; Figure 6D,F) and increased with the non-selective β-AR agonist isoproterenol (10 µM [49]; 3.9 ± 1.2 Hz vs. 2.9 ± 1.1 Hz vs. in vehicle; *n* = 7; *p* = 0.02, Wilcoxon test; Figure 6G). ChIs from DAT(dT) mice responded similarly to the β1-AR ligands. ChI firing decreased with propranolol (3.3 ± 0.7 Hz vs. 3.9 ± 0.5 Hz in vehicle; *n* = 9; *p* = 0.02, Wilcoxon test; Figure 6D,H) and increased with isoproterenol (6.3 ± 0.7 Hz vs. 2.9 ± 0.77 Hz in vehicle; *n* = 8; *p* = 0.008, Wilcoxon test; Figure 6I). Propranolol reduced the firing frequency of ChIs in DAT(dT) mice by 0.6 ± 0.3 Hz (23 ± 9%) and by 1.2 ± 0.5 Hz (21 ± 8%) in WT(dT) mice (*p* = 0.3, *t*-test; Figure 6J). Isoproterenol increased the firing frequency of ChIs in DAT(dT) mice by 3.4 ± 0.7 Hz (214 ± 66%) and by 1.1 ± 0.3 Hz (52 ± 17%) in WT(dT) mice (*p* = 0.01, *t*-test; Figure 6K). The results show that β-AR modulate ChIs in the DL striatum and suggest that progressive DA deficiency may enhance the excitatory response of ChIs to β-AR agonists.

## 4. Discussion

Parkinsonism is a disabling condition that is produced by a reduction in DA availability. While L-Dopa remains the mainstay of treatment, evidence from human and animal studies suggests that DA deficiency creates a subsequent decline in ACh availability that contributes to the motor deficit [13,19,20]. LID is a major barrier to treatment in some patients with dopa-responsive dystonia [6,7], and eventually occurs in 30–80% of PD patients [3,4,5]. The cause of LID remains unclear, but recent evidence points toward the imbalance in the ACh to DA ratio [12,13,14,15]. If so, targeting the ChI, the main source of striatal ACh might prevent or alleviate this secondary movement disorder.

We used a new transgenic mouse as a model for Parkinsonism. While no animal model replicates the pathology of PD in humans [50], the exposure of *Slc6a3^DTR/+^* mice to dT produces a progressive reduction in striatal DA—by ~90% over 4 weeks—and a decline in motor control [13]. We found that daily treatment with L-Dopa and benserazide rescued the DAT(dT) mice, increasing their survival rate from 2 to 4 weeks [13] to greater than 17 weeks. Weight and locomotor activity were preserved, while performance on the balance beam and rotarod was only marginally depressed when compared with the controls. Under the treatment parameters, a subset of mice (30%) developed LID, characterized by abnormal repetitive limb movements. This is consistent with estimates of LID in humans [3,4,5], but it is possible that the β-AR blocker prevented the emergence of LID in some of the mice. These responder mice also manifested higher locomotor ambulations and lower motor skill learning and coordination on behavioral testing, consistent with the development of LID in humans, where a higher incidence and/or early development of LID is related to the severity and duration of disease [9,32].

LID observed in DAT(dT) mice was more symmetric with relative lack of spinning and unilateral movements that may be seen following unilateral ablation of striatal DA [39]. This LID was suppressed by the higher dose of the β-AR blocker propranolol, suggesting its potential therapeutic use in PD. Consistent with prior studies [47,51,52], propranolol reduced LID without adversely affecting either motor performance or exploratory behavior in the open field, indicating its anti-dyskinetic effect is not due to a reduction in general motor activity. Interestingly, we observed an increase in the locomotor ambulations of DAT(dT) non-responder mice, relative to WT(dT) controls, that was suppressed by propranolol. The reduction of locomotor activity with propranolol was less evident in DAT(dT) responder mice. However, while there was a slight (non-significant) increase in limb dyskinesia during the washout, we observed a significant increase in locomotor ambulations in both responder and non-responder mice during this period, indicating an additional anti-dyskinetic effect of propranolol. Propranolol has been shown to reduce LID in monkeys [52] and rats [47,51] following 6-OHDA. Propranolol also reduced LID by 40% in a small clinical trial [31]. Propranolol may ameliorate LID in a dose-dependent way without affecting L-Dopa’s efficacy, while high doses may reduce LID at the expense of exacerbating Parkinsonian symptomatology [47,51,52].

Our study demonstrates the development of LID in an L-Dopa-responsive mouse model of Parkinsonism with progressive DA deficiency. In *Slc6a3^DTR/+^* mice, dT targets only cells that contain the DAT and progressively destroys both axon terminals in the striatum and DA-producing cell bodies in the substantia nigra [13,33]. This work confirms that spontaneous ChI firing is lower in DA-deficient mice [13], that β-ARs modulate ChI in the DL striatum [25,26], and it demonstrates the utility of targeting adrenergic receptors to restore the balance between ACh and DA. A limitation of the study is our use of young mice rather than the older rodents that have typically been used to analyze the effects of L-Dopa in Parkinsonian mice. We used adolescent mice at time points that corresponded to our earlier work in the DA-depleted dorsal striatum [13,38,53] and may better represent juvenile forms of PD. The electrophysiology data in the slice preparation may differ from plasticity that occurs following treatment with L-Dopa in vivo, presenting an avenue for future research. WT mice without dT treatment were not used as a control, as prior data indicated that the dose of dT used here has no effect on the behavior or physiology of non-transgenic mice [13] and L-Dopa does not promote LID in WT mice [40]. The dose of L-Dopa was similar to that used to rescue DA-deficient transgenic mice that lacked tyrosine hydroxylase [38] and not specifically optimized to induce LID. The doses of propranolol were chosen to allow comparison with other studies [47]. Higher doses might promote further improvement in LID but possibly at the expense of motor function [47,51,52]. We would predict that a β-AR agonist would heighten ChI firing and increase LID in DAT(dT) mice, but β-AR agonists that cross the blood-brain barrier are generally unavailable due to their toxicity in vivo.

**Figure 7:**
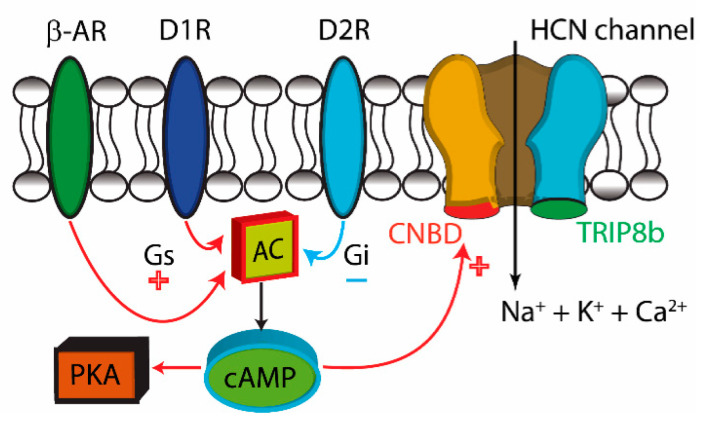
β-adrenergic receptors (β-ARs) enhance the spontaneous firing of ChIs through hyperpolarization-activated cation (HCN) channels. The HCN channel opens when the cell becomes hyperpolarized after each action potential and the flow of cations through the channel and into the cell provides rapid depolarization of the ChI, leading to repetitive firing. D1 (D1R) and D2 receptors (D2R) are coupled to the G-protein, adenylyl cyclase (AC) signal transduction pathway that modifies the rate of ChI firing via cAMP, independently of PKA [26,27]. β1-ARs and β2-ARs also enhance cAMP [25,30], which binds to the HCN cyclic nucleotide-binding domain (CNBD) [27] to reduce inhibition of HCN channel gating. HCN channels consist of four α-subunits and the β-subunit, TRIP8b, an auxiliary HCN tetratricopeptide repeat containing Rab8b-interacting protein [27]. TRIP8b modulates HCN surface expression and negatively affects activation of CNBD by cAMP [55].

Monoamine action plays an essential role in striatal physiology and motor control [11,22]. Although research has often focused on DA, adrenergic receptors also contribute to striatal regulation [25,26,54]. Our data show that β-ARs modify the tonic firing rate of striatal ChIs in the DL striatum. β-AR are known to excite ChIs through a 3’,5’-cyclic adenosine monophosphate (cAMP)-dependent, but protein kinase A (PKA)-independent pathway [26], a similar mechanism used by DA receptors to modulate the tonic firing of ChIs that is driven by HCN channels [27] (Figure 7). Our data shows that prolonged DA deficiency induces a larger increase in ChI firing by the β-AR agonist isoproterenol. Prolonged DA deficiency is also known to heighten the response of D1R agonists on ChIs [13], suggesting sensitivity changes in catecholamine receptors or down-stream alterations in adenylyl cyclase, cAMP, or components of the HCN channel. However, additional mechanistic studies will be required as the increase in cAMP production also leads to diverse intracellular changes via the rapid phosphorylation of cAMP response element binding protein (CREB), a transcription factor implicated as a molecular switch underlying long-term changes in brain function [25].

While β-ARs are located on cells throughout the brain, the antagonist’s effect on LID likely occurs within the striatum since an intrastriatal infusion of propranolol acutely reduces LID [51]. Propranolol seems to have little effect on dyskinesia produced by D1 or D2 receptor agonists [47], suggesting a presynaptic mechanism such as modulation of L-Dopa-mediated DA efflux [47], perhaps mediated through ACh regulation of DA release [55,56]. Therefore, in addition to its anticholinergic effect, propranolol’s anti-dyskinetic properties may also be mediated via attenuation of L-Dopa-induced extra-physiological efflux of DA [47,55], thereby acting to improve the ACh and DA ratio during treatment of DA deficiency. Amantadine has been used most frequently in the treatment of LID and likely modifies the ACh to DA ratio. While being a weak N-Methyl-D-aspartate (NMDA) receptor antagonist [57], this anticholinergic drug inhibits nicotine currents [58] and modifies DA release [59,60]. This report provides additional evidence that β-ARs may be a potential target for novel treatment of LID. While our study has focused on propranolol, the anti-dyskinetic effect of this drug is likely related to its efficacy as a β-blocker that promotes a secondary anticholinergic action. Propranolol has been traditionally used to treat a variety of neurological and systemic symptoms [29,30], but with broad anticholinergic properties, propranolol may not be suitable for the elderly. However, centrally-acting β-blockers or drugs that might specifically target β-ARs or other G-protein coupled receptors on ChIs might also be expected to improve LID. By reducing intracellular cAMP, these drugs would diminish the abnormal firing rate of ChIs and reduce the heightened release of ACh that occurs in Parkinsonism following treatment with L-Dopa.

## Figures and Tables

**Table 1:** Dyskinesia scoring.

**General Dyskinesia**	
0	Normal behavior
1	Twitching with low or no movement
2	Shaking arms coupled with low or no movement
3	Perched on hind legs while craning to one side or abnormal arm posture
4	(3) but for longer than three seconds
**Limb Dyskinesia**	
0	Normal behavior
1	Moving a limb repeatedly
2	≥2 limbs move slowly
3	≥2 limbs move quickly
4	Waving limbs while standing

**Table 2:** Weekly schedule of treatment and behavioral testing.

Monday	Treatment
Tuesday	Treatment
Wednesday	Pre-treatment habituation and monitoring (90 min); treatment; locomotor and behavioral monitoring (120 min)
Thursday	Pre-treatment habituation and monitoring (90 min); treatment; locomotor and behavioral monitoring (120 min)
Friday	Rotarod and balance beam on treatment weeks 1, 10, 13, 16, and 18; pre-treatment habituation (90 min); treatment; locomotor and behavioral monitoring (120 min)
Saturday	Treatment
Sunday	Treatment

## Supplementary Materials

The following are available online at https://www.mdpi.com/2076-3425/10/12/903/s1: Supplemental video 1. The video shows the typical behavior of a WT control mouse in an open field. Supplemental video 2. The video demonstrates the behavior of a DAT(dT) responder mouse on treatment week 11. The mouse had received 11 weeks of L-Dopa with benserazide and one week of low-dose propranolol. Supplemental video 3. The video demonstrates the same mouse shown in Supplemental video 1 on treatment week 15, following high-dose propranolol. This mouse had received 15 weeks of L-Dopa with benserazide, three weeks of low-dose propranolol, and two weeks of high-dose propranolol.
