# Supplementary figures and images for: Propranolol Relieves L-Dopa-Induced Dyskinesia in Parkinsonian Mice
Source: Brain Sci. 2020 Nov 24;10(12):903. doi: 10.3390/brainsci10120903 (PMC7760026; doi:10.3390/brainsci10120903)

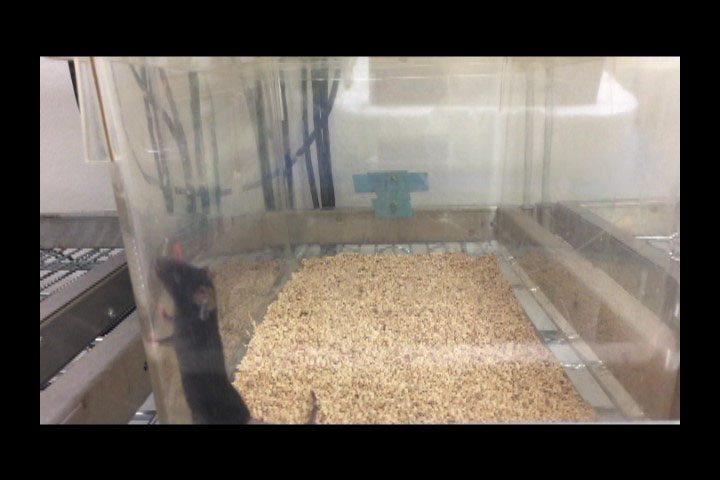

Supplement: Supplementary file 1 [file brainsci-10-00903-s001.zip › Supp Movies/SuppMovie_S1 thumbnail.jpg]

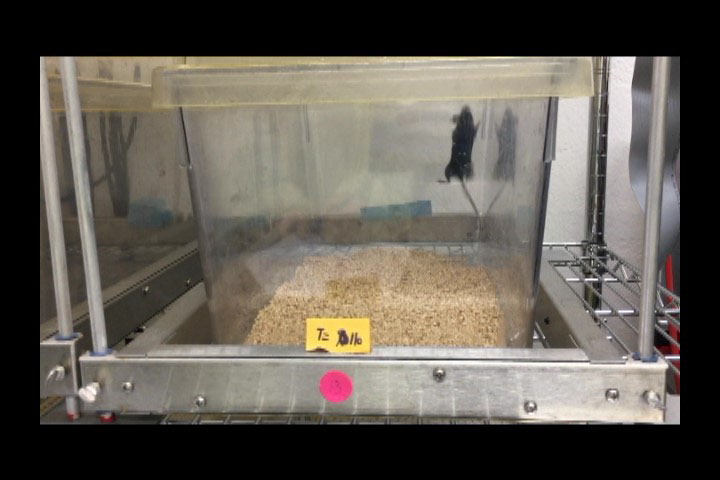

Supplement: Supplementary file 1 [file brainsci-10-00903-s001.zip › Supp Movies/SuppMovie_S2 thumbnail.jpg]

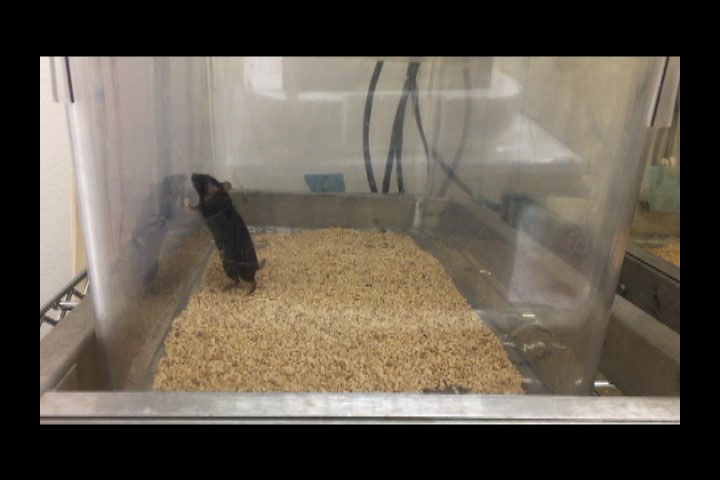

Supplement: Supplementary file 1 [file brainsci-10-00903-s001.zip › Supp Movies/SuppMovie_S3 thumbnail.jpg]
